# COVID-19 and Organ Transplantation?

**Published:** 2020

**Authors:** A. Zomorrodi

**Affiliations:** 1 *Professor of urology and kidney transplant surgeon, head of organ transplantation and urology department, Tabriz medical science university *

**Dear Editor,**

I read with interest the letter of Joob and Wiwanitkit on COVID-19 and organ transplantation, published recently in the IJOTM [1]. Herein, I would like to comment on it. Viral infection is one of the most common infections after kidney transplantation; it does not occur only during the first days; it occurs in later days too. Viral infection has two main effects on recipients—directly by the virus, which induces fever and leukopenia, and indirectly through the release of cytokines [2, 3]. Therefore, recipients, health care workers, and family members of the recipients are still advised to receive vaccination against influenza before and after the transplantation [4]. Recipients should also receive other relevant medications, especially the immunosuppressant agents. So it is very soon to jump to the conclusion made in their letter that COVID-19 is less common in transplant recipients and that it is associated with a good prognosis [1]; it needs more careful studies. In another letter published in the New England Journal of Medicine, 28% of kidney recipients with COVID-19 died [5]; the mortality was 1% among normal people diagnosed with COVID-19. The mortality was very high in our department too. We had four kidney transplant recipients with COVID-19; two of them died. The most common symptoms were fever and diarrhea [5]. Definitive conclusion needs extensive study in long time. 
