# Supplementary material for: COVID-19 and Organ Transplantation?
Source: Int J Organ Transplant Med. 2020;11(3):143–4. (PMC7471612)
Supplement: Related texts

# REPLY

**Published:** 2020

**Authors:** B. Joob, V. Wiwanitkit

**Affiliations:** 1 *Sanitation 1 Medical Academic Center, Bangkok Thailand *; 2 *Honorary Professor, Dr DY Patil University, Pune, India *

We appreciate the comments on our letter [1]. As we have already mentioned in our first correspondence, there should be more data on COVID-19 among organ transplant patients. The mortality in COVID-19 is considered higher than other respiratory virus infections and the mortality rate might be varied in different settings based on demographic characteristics, quality of care, comorbidity, stage of the disease at initial presentation and outbreak density. For example, an elderly with diabetes might have increased chance of mortality regardless of having organ transplantation [2]. The effect of immunosuppressive drug is also an important issue to be discussed. For example, cyclosporine, a classical immunosuppressive agent, was previously mentioned for its effect against corona virus infection [3]. Finally, apart from renal transplantation, there are transplantations of other organs where we have still no data on COVID-19. For example, it is still an interesting issue about the mortality among COVID-19 patients with hematological stem cell transplantation [4]. The study on kidney transplant recipients with COVID-19 might show the high mortality, however, it has to be compared with the background situation in that setting. Also, it should address for the use of immunosuppressive drugs in that setting. As we have stated in our first correspondence, the available data are not enough to make a solid conclusion. 

## CONFLICTS OF INTEREST:

None declared.

## References

[1] Joob B, Wiwanitkit V (2020). letter to editor, COVID-19 and Organ Transplantation. Int J Organ Transplant Med.

[2] Xu G, Yang Y, Du Y (2020). Clinical Pathway for Early Diagnosis of COVID-19: Updates from Experience to Evidence-Based Practice. Clin Rev Allergy Immunol.

[3] de Wilde AH, Raj VS, Oudshoorn D (2013). MERS-coronavirus replication induces severe in vitro cytopathology and is strongly inhibited by cyclosporin A or interferon-α treatment. J Gen Virol.

[4] Ardura MI, Hartley DM, Dandoy C (2020). Addressing the Impact of the Coronavirus Disease (COVID-19) Pandemic on Hematopoietic Cell Transplantation: Learning Networks as a Means for Sharing Best Practices. Biol Blood Marrow Transplant.
